# Minor physical anomalies in neurodevelopmental disorders: a twin study

**DOI:** 10.1186/s13034-017-0195-y

**Published:** 2017-11-28

**Authors:** Lynnea Myers, Britt-Marie Anderlid, Ann Nordgren, Charlotte Willfors, Ralf Kuja-Halkola, Kristiina Tammimies, Sven Bölte

**Affiliations:** 1 Department of Women’s and Children’s Health, Center of Neurodevelopmental Disorders (KIND), Karolinska Institutet & Center for Psychiatry Research, Stockholm County Council, Stockholm, Sweden; 2 Department of Molecular Medicine and Surgery, Karolinska Institutet, Stockholm, Sweden; 3 Department of Clinical Genetics, Karolinska University Hospital, Stockholm, Sweden; 4 Department of Medical Epidemiology and Biostatistics, Karolinska Institutet, Stockholm, Sweden; 5 Department of Women’s and Children’s Health, Center of Neurodevelopmental Disorders (KIND), Karolinska Institutet & Child and Adolescent Psychiatry, Center for Psychiatry Research, Stockholm County Council, Gävlegatan 22B, 113 30 Stockholm, Sweden

**Keywords:** Autism, ASD, ADHD, Minor physical anomalies, Neurodevelopmental disorders

## Abstract

**Background:**

Minor physical anomalies (MPAs) are subtle anatomical deviations in one’s appearance and may suggest altered embryogenesis. MPAs have been shown to be more common in neurodevelopmental disorders (NDDs) compared with typical development. Still, further studies are needed on MPAs in NDDs, especially using twins to adjust for confounding familial factors.

**Methods:**

Clinical assessments were conducted on 116 twins (61 NDD, 55 controls) from 51 monozygotic and 7 dizygotic pairs to examine MPAs and their association with DSM-5 defined NDDs. Additionally, the relationship between the number of MPAs within twins by zygosity was investigated.

**Results:**

Within the cohort sample, a specific association was found between MPAs and autism spectrum disorder (ASD) diagnosis (crude odds ratio = 1.29, p = .047; adjusted odds ratios = 1.26–1.33, adjusted p values = .032–.073) and autistic traits (crude β = 3.02, p = .002; adjusted β = 2.28, p = .019), but not NDDs in general or ADHD, nor within-pairs. Identified MPAs in ASD included overweight, hypermobility, pes planus, straight eyebrows, vision impairment, arachnodactyly/long toes, long eyelashes, and microtia. The number of MPAs within all monozygotic pairs was highly correlated (r = .88, p < .001).

**Conclusion:**

MPAs are more frequent in participants with ASD and may be influenced by genetics. The value of MPAs for (early) detection should be further explored, as they might index individuals at increased risk for ASD in particular.

**Electronic supplementary material:**

The online version of this article (10.1186/s13034-017-0195-y) contains supplementary material, which is available to authorized users.

## Background

Minor physical anomalies (MPAs) are defined as subtle, abnormal morphological features, such as deviations in morphology of the head, eyes, ears, mouth, hands, and feet [1]. As the brain and skin are derived from the same neuroectodermal layer during early fetal development, MPAs may mirror altered brain development [2]. Therefore, the presence of multiple MPAs could suggest the possibility of an underlying genetic and/or environmental perturbation affecting embryogenesis.

Neurodevelopmental disorders (NDDs) emerge in early childhood and cause persistent impairment in cognitive, social, academic, and/or occupational functioning. NDDs include intellectual disability (ID), communication disorders, autism spectrum disorder (ASD), attention-deficit/hyperactivity disorder (ADHD), specific learning disorders, and motor disorders and there is substantial overlap among NDDs [3]. For the purposes of this study, participants were diagnosed with NDDs according to DSM-5 and could have one or more NDDs based on the outcomes of their diagnostic assessment.

MPAs are common in the general population [4, 5]. In NDDs, the presence of MPAs has been studied predominantly in ASD, ADHD, and ID [4, 6–9]. Multiple MPAs have been found in approximately 20% of individuals with ASD [1, 10]. Ozgen et al. [9] conducted a meta-analysis comparing effect sizes of seven studies exploring MPAs in ASD and found higher frequencies of MPAs in individuals with ASD compared to typically developing (TD) controls. Only one study has examined MPAs in twins with ASD [11] and found that affected twins exhibited more MPAs than their co-twins. However, the assessment only covered a few MPAs and the findings were minimally described. Meanwhile, studies examining the association between ADHD and MPAs demonstrate mixed results. One found no association between MPAs and ADHD, but with ID [6], while others found higher rates of MPAs in children diagnosed with ADHD [12, 13]. Research is lacking on the relationship between MPAs and NDDs as an overarching diagnostic entity as defined by the DSM-5.

Ozgen et al. [4, 9] addressed limitations in studies to date on MPAs in children with ASD. They noted that studies did not use standardized instruments to determine diagnosis, had participants with varying ethnic backgrounds, used physical examinations not originally designed for MPA assessment, lacked controls, examined small samples, failed to report interrater agreement, and did not account for the effects of gender and intelligence (IQ) on MPA scores. Further studies are needed that address these limitations, especially using twins to adjust for confounding familial factors.

The objective of the current study was to investigate MPAs in carefully characterized participants with NDDs in a rare sample of monozygotic (MZ) and dizygotic (DZ) twins concordant or discordant for the conditions and TD control pairs. We aimed to examine (i) the frequency and most common MPAs in relation to diagnostic status, (ii) the association between MPAs and NDD diagnoses, (iii) MPAs by twin pair zygosity, and (iv) MPA differences in discordant pairs. We expected an excess of MPAs in individuals with NDD diagnoses compared with TD controls. Moreover, we predicted that affected twins from MZ NDD-discordant pairs would have more MPAs than their unaffected co-twins. Lastly, we expected that MZ pairs overall, compared to DZ pairs, would present with similar amounts and types of MPAs.

## Methods

### Sample

Participants were part of the Roots of Autism and ADHD Twin Study in Sweden, described elsewhere in detail [14], from August 2011 to November 2013. They were recruited through the Child and Adolescent Twin Study in Sweden via advertisements in journals of national interest organization, referrals from clinical units (e.g., child psychiatry, habilitation centers), and the Swedish patient registry. A total of 116 twins, representing 51 MZ pairs and seven DZ pairs underwent diagnostic, behavioral, and MPA assessments. The study was approved by the Regional Swedish Ethical Review Board.

### Diagnostic and behavioral assessments

Participants were either TD or diagnosed as having a NDD or for some, more than one NDD (indicating co-morbidity of diagnoses), using a consensus process with several experienced clinicians according to DSM-5 criteria corroborated by information from standardized instruments: Autism Diagnostic Observation Schedule (ADOS-2); Autism Diagnostic Interview-Revised (ADI-R); Kiddie Schedule for Affective Disorders and Schizophrenia (K-SADS); and Diagnostic Interview for ADHD in Adults (DIVA 2.0) (see Additional file 1: Table S1 for instrument details). IQ testing was performed with the following measures: Wechsler Adult Intelligence Scale-IV (WAIS-IV); Wechsler Intelligence Scale for Children-IV (WISC-IV); Leiter International Performance Scale-Revised; and the Peabody Picture Vocabulary Test (PPVT-4). Autistic traits were measured with the Social Responsiveness Scale-2 (SRS-2). As recommended for research settings, the SRS-2 total raw score was used, with increasing scores (0–195) indicating more severe traits. The twin pairs were categorized as either NDD-concordant (i.e., both twins meeting criteria for NDD diagnosis), NDD-discordant (i.e., only one twin in pair meeting criteria for NDD diagnosis), or TD (i.e., neither twin meeting criteria for NDD diagnosis). Structural magnetic resonance imaging (sMRI) was conducted to identify any gross brain alterations. Blood and saliva were collected to confirm zygosity. Individuals reported to have known genetic syndromes (e.g., Down) were excluded from the study to prevent confounding variables, similar to previous research [4, 7].

### MPA assessment

A checklist (Additional file 2: Table S2) containing a total of 179 anomalies for males (24 body regions) and 171 for females (23 body regions) was used in the study. The checklist was developed and utilized by two experienced clinical geneticists (authors BMA, AN) based on the London Dysmorphology Database, Elements of Morphology [15], and long-standing clinical expertise. Standardized terms for MPAs are based on the Human Malformation Terminology from the Elements of Morphology [15]. The checklist includes MPAs, as well as anomalies that are commonly assessed for in physical exams (e.g., overweight, vision impairment, etc.). Vision impairments included myopia, hyperopia, colorblindness, astigmatism, etc. and 35% of participants with impairments had corrective lenses. Common phenotypic variants from the original checklist were removed if they were present in more than 4% of the European population [4, 16]. The MPA assessments took approximately 1 h to complete for each pair. The assessments were completed by either one or both clinical geneticists, with the majority completed by both. Any disagreements related to the presence of MPAs were resolved on the spot. Participants received a score of “1” for the presence of every MPA on the checklist, as well as a score of “1” for any clinically relevant findings on the sMRI (as read by a radiologist; 93% had data). Length and weight were measured by a research nurse and corrections were made to the overweight and underweight items on the checklist for each participant based on their actual body mass index (BMI) for individuals 20 years of age or older and BMI-for-age for individuals less than 20 years old. Additionally, a physician trained in dysmorphology assessed photographs of 28 twin pairs from the study to establish interrater agreement on all items mutually visible on both the in-person MPA assessment and through photographs (90 items).

### Statistical analysis

Only pairs with full MPA assessments were included in the analyses (n = 58 pairs) to allow for statistical procedures making comparison within twin pairs. Two pairs of twins were removed a priori due to one twin with TD and another twin with ASD not consenting to the MPA assessment. Medians and interquartile ranges are reported for MPA scores by diagnosis and concordance type and means and standard deviations are used for demographic data related to the participants. To adjust for the clustering in pairs, a conditional regression model was fitted using generalized estimating equation (GEE) analyses [17] for assessments of the association between the number of MPAs and (i) all NDD diagnoses (including ASD and ADHD), (ii) ASD only, and (iii) ADHD only. The number of MPAs was the independent variable and the diagnostic category or traits were the dependent variables. Two models were fitted: first, a linear regression model for estimates of associations in the *cohort* with clustered standard errors accounting for the twin correlation, and next, a conditional regression model for estimates of association *within*-*pairs* after adjusting for factors shared within twins like genetics and environment. The *cohort* linear model assesses for associations between variables, but accounts for the dependence of participants as they are twins and have more in common with each other than other samples. The *within*-*pairs* conditional regression model estimates the association among variables of interest using the differences in outcome values (in this case, the MPA scores) in monozygotic pairs, to account for their genetic relatedness and other shared factors. Previous publications recommend controlling for gender and IQ in analyses of MPAs [4, 9], but only IQ was controlled for in the analyses as there was no association in this sample between gender and MPAs. Additional analyses explored the cross-trait association between the MPA score in one twin with the (i) total raw SRS-2 score and (ii) IQ for the other twin to explore the genetic correlation of the measures. Results for the *cohort* and *within*-*pairs* GEE analyses described above are reported as odds ratios (OR) and/or β estimates with 95% confidence intervals (CI). Statistics were calculated with SPSS version 24 and R version 3.3.2.

## Results

### Participants

Of the 116 participants in the study, 53% (n = 61) had a NDD diagnosis and 47% (n = 55) were TD. The percentage of specific NDD diagnoses in the sample were the following: 24% (n = 28) had a diagnosis of ASD, 32% (n = 37) ADHD, 8% (n = 9) ID, and 26% (n = 30) with other NDDs (e.g., motor, communication, or specific learning disorders). Fifty-seven percent (n = 66; 62% with NDDs) were male, and 43% (n = 50; 40% with NDDs) were female. Ages at examination ranged from 9 to 23 years (mean = 14.05, standard deviation = 3.40) (see Table 1). Details related to participants’ parents can be found in Additional file 3: Table S3.

**Table 1:** Demographic information on the sample by diagnosis and concordance for 58 twin pairs

	TD	ASD			ADHD			NDD		
	Concordant	Concordant	Discordant		Concordant	Discordant		Concordant	Discordant	
Number of pairs	19	8	12		13	11		22	17	
Zygosity by pairs (MZ:DZ)	18:1	8:0	10:2		12:1	7:4		19:3	14:3	
Sex by twin (female:male)	21:17	6:10	9:15		8:18	8:14		10:34	19:15	
Age, mean	15.26	13.88	13.50		13.69	13.00		14.14	12.59	

			Affected twin	Co-twin		Affected twin	Co-twin		Affected twin	Co-twin
IQ, mean	103.37	83.44	91.25	97.92	86.85	101.27	97.27	86.52	94.82	97.94
(SD, range)	(13.80, 81–138)	(16.73, 58–110)	(18.78, 69–121)	(17.20, 65–121)	(14.56, 64–110)	(11.22, 84–121)	(9.98, 85–121)	(15.72, 58–115)	(16.16, 70–121)	(11.50, 77–121)
SRS-2, total raw mean	18.47	89.81	87.75	36.42	79.77	56.91	32.18	70.68	57.53	25.82
(SD, range)	(14.58, 0–65)	(33.30, 38–141)	(27.02, 41–130)	(23.29, 8–93)	(37.27, 33–142)	(34.35, 15–117)	(15.57, 14–55)	(38.10, 14–142)	(33.78, 3–117)	(13.19, 8–51)
# Participants with other diagnoses										
ASD: n (%)		16 (100.00)	12 (100.00)	0 (0.00)	12 (46.15)	4 (36.36)	1 (9.09)	19 (43.18)	9 (52.94)	
ADHD: n (%)	0 (0.00)	11 (68.75)	5 (41.67)	2 (16.67)	26 (100.00)	11 (100.00)	0 (0.00)	30 (68.18)	7 (41.17)	0 (0.00)
ID: n (%)	0 (0.00)	4 (25.00)	1 (8.33)	1 (8.33)	4 (15.38)	0 (0.00)	0 (0.00)	8 (18.18)	1 (5.88)	0 (0.00)
Other NDD: n (%)	0 (0.00)	7 (43.75)	5 (41.67)	1 (8.33)	12 (46.15)	4 (36.36)	3 (27.27)	23 (52.27)	7 (41.17)	0 (0.00)

Demographic findings for the sample by TD or diagnosis and concordance of diagnosis. Means, standard deviations, and ranges are used to describe demographic data related to the participants. Participants may be represented in more than one column in the table if they have concurrent diagnoses (e.g., diagnosis of both ASD and ADHD). Examples of other NDDs include communication disorder, specific learning disorder, motor disorder, or other neurodevelopmental disorders

*MZ* monozygotic, *DZ* dizygotic, *SD* standard deviation, *IQ* intelligence quotient, *ASD* autism spectrum disorder, *ADHD* attention-deficit/hyperactivity disorder, *ID* intellectual disability, *NDD* neurodevelopmental disorder

### Interrater agreement for MPA checklist

The interrater agreement between the two sets of raters ranged from 70.4 to 100% across the MPAs. The MPAs with the lowest agreement were downslanted palpebral fissure (70.4%), long eyelashes (70.4%), and straight eyebrows (74.1%). There were numerous MPAs with 100% agreement, including ectrodactyly of the hands and feet, broad thumbs, prominent heels, wide mouths, and hirsutism.

### MPAs in NDDs

**Fig. 1:**
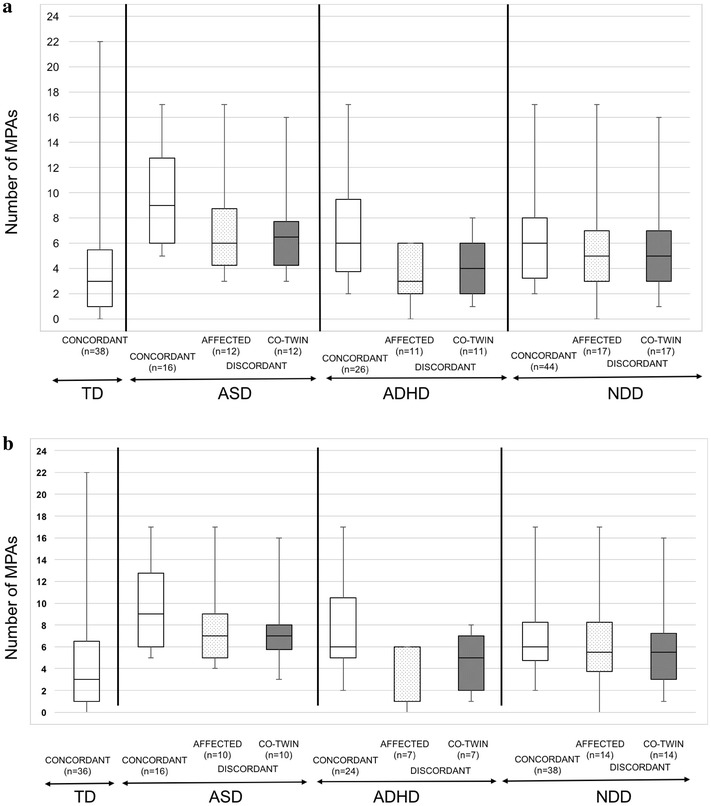
Box plots illustrating the median number of MPAs in twins, as well as 25th and 75th interquartile range and minimum and maximum values based on TD or diagnosis and concordance of diagnosis for **a** the cohort sample and **b** MZ twins only. Twins with concordant ASD had the highest median number of MPAs (Md = 9) followed by discordant ASD co-twins. Those with TD had the lowest number of MPAs (Md = 3). These trends are similar for the MZ only twins with the concordant ASD twins having the highest number of MPAs (Md = 9), followed by a Md = 7 MPAs for discordant ASD affected twins and co-twins. *MPAs* minor physical anomalies, *TD* typical development, *ASD* autism spectrum disorder, ADHD attention–deficit/hyperactivity disorder, NDD neurodevelopmental disorder

Twin pairs concordant for NDDs had a median of six MPAs, followed by a median of five MPAs for NDD-discordant co-twins and affected twins, respectively (Fig. 1a). In comparison, pairs concordant for TD had a median of three MPAs. These trends were similar for MZ twins (Fig. 1b). The most common MPAs in participants with NDDs were overweight (33%), arachnodactyly/long toes (31%), hypermobility (26%), straight eyebrows (25%), and vision impairment (21%; 46% of these with corrective lenses). In comparison, microtia (24%) and arachnodactyly/long toes (22%) were the only MPAs present in greater than 20% of participants with TD.

No significant association was found between a diagnosis of any NDD and the number of MPAs in the cohort (OR = 1.09, 95% CI = .94–1.27, p = .256). Since there was an association between IQ and MPAs in the cohort (β = − .95, SE = .32, 95% CI = − 1.59 to − .32, p = .003), in that for every MPA, the IQ score decreased by about one point, we controlled for IQ in the model testing the association between any NDD diagnosis and MPA, and the results did not change (OR = 1.05, 95% CI = .89–1.24, p = .592). Conversely, a cross-trait analysis comparing the MPA score from one twin to the IQ in the co-twin showed a strong association (β = − .88, 95% CI = − 1.52 to − .24, SE = .327 p = .007; Additional file 4: Table S4). The within-pairs association between NDD diagnosis and MPAs was neither significant for all participants (OR = 1.10, 95% CI = .46–2.65, p = .832), nor MZ twins only (OR = 1.11, 95% CI = .44–2.82, p = .824) (Additional file 5: Table S5).

### MPAs in ASD and in relation to autistic traits

Twins pairs concordant for ASD had descriptively the highest median (Md) number of MPAs (Md = 9), followed by a median of six-and-a-half and six MPAs for ASD-discordant co-twins and affected twins, respectively (Fig. 1a). These trends were similar for MZ twins (Fig. 1b). The most common MPAs in participants with ASD included overweight (39%), hypermobility (36%), pes planus (29%), straight eyebrows (29%), vision impairment (25%; 29% of these with corrective lenses), arachnodactyly/long toes (25%), long eyelashes (21%), and microtia (21%).

There was an association between a diagnosis of ASD and MPAs (OR = 1.29, 95% CI = 1.00–1.66, p = .047). The association remained when controlling for ADHD (OR = 1.29, 95% CI = 1.02–1.63, p = .032), other NDD diagnoses (OR = 1.33, 95% CI = 1.05–1.67, p = .016), but not IQ (OR = 1.26, 95% CI = .98–1.61, p = .073). The within-pairs association for ASD was neither significant for all participants (OR = 1.28, 95% CI = .60–2.75, p = .529), nor MZ twins only (OR = 1.42, 95% CI = .63–3.19, p = .398) (Additional file 5: Table S5).

Due to the association between the number of MPAs and a clinical diagnosis of ASD, further analyses were conducted to examine if the association held when the number of MPAs was analyzed in relation to the severity of autistic traits. Using the linear regression model, there was an association between MPA scores and SRS-2 scores in the entire sample (β = 3.02, SE = .98, 95% CI = 1.09–4.94, p = .002), indicating that for every MPA, there was an approximately three-point increase in SRS-2 scores. The association remained when controlling for IQ (β = 2.28, SE = .97, 95% CI = .37–4.18, p = .019), but neither in the within-pairs analysis (β = 1.49, SE = 1.80, 95% CI = − 2.04 to 5.02, p = .409), nor MZ only twins (β = 2.11, SE = 1.85, 95% CI = ^−^1.51–5.73, p = .254) (Table 2 and Additional file 5: Table S5). A cross-trait analysis comparing the MPA score from one twin in each pair to the total SRS-2 raw score showed a strong association (β = 3.34, 95% CI = 1.39–5.30, SE = 1.00, p < .001; Additional file 4: Table S4).

**Table 2:** Selected cohort and within-pairs estimates between mpas and categorical diagnoses/dimensional variables (Autistic Traits, IQ)

Categorical diagnoses		Cohort estimate				Within-pairs conditional estimate				MZ only within-pair conditional estimate			
		β	OR	95% CI (OR)	p value	β	OR	95% CI (OR)	p value	β	OR	95% CI (OR)	p value
ASD and MPAs													
No adjustments		.25	1.29	1.00–1.66	.047	.25	1.28	.60–2.75	.529	.35	1.42	0.63–3.19	.398
Adjusted for IQ		.23	1.26	.98–1.61	.073	.16	1.18	.55–2.54	.673	.55	1.73	.47–6.45	.413
Adjusted for ADHD		.25	1.29	1.02–1.63	.032	1.00	2.71	.84–8.80	.097	1.00	2.71	.83–8.81	.097
Adjusted for other NDDs		.28	1.33	1.05–1.67	.016	1.00	2.71	.84–8.80	.097	1.00	2.71	.83–8.81	.097

Dimensional variables		Cohort estimate				Within-pairs conditional estimate				MZ only within-pair conditional estimate			
		β	SE	95% CI (β)	p value	β	SE	95% CI (β)	p value	β	SE	95% CI (β)	p value
IQ and MPAs													
No adjustments		− .95	.32	− 1.59 to − .32	.003	.30	.69	− 1.04 to 1.64	.665	− .15	.67	− 1.46 to 1.17	.828
SRS-2 and MPAs													
No adjustments		3.02	.98	1.09 to 4.94	.002	1.49	1.80	− 2.04 to 5.02	.409	2.11	1.85	− 1.51 to 5.73	.254
Adjusted for IQ		2.28	.97	.37 to 4.18	.019	1.78	1.83	− 1.82 to 5.37	.332	1.88	1.68	− 1.41 to 5.17	.263

Cohort and within-pairs associations between ASD, autistic traits and the number of minor physical anomalies (MPAs). The *cohort* model estimates associations between variables while accounting for the twin correlation; the *within*-*pairs* model estimates associations after adjusting for factors shared within twins like genetics and environment. ADHD or other NDD diagnoses, as well as IQ, were used as covariates in the each of the models

*MPAs* minor physical anomalies, *MZ* monozygotic, *IQ* intelligence quotient, *ASD* autism spectrum disorder, *ADHD* attention–deficit/hyperactivity disorder, *ID* intellectual disability, *NDD* neurodevelopmental disorder, *SRS-2* Social Responsiveness Scale-2

We also examined the presence of MPAs in all seven MZ ASD-discordant twin pairs in the sample. Of note, the overall number of MPAs was similar within pairs or even higher at times for the unaffected co-twin, but two out of seven ASD affected twins had scoliosis, which was absent in their co-twins (Additional file 6: Table S6).

### MPAs in ADHD

Twin pairs concordant for ADHD had a median of six MPAs each, followed by a median of four and three MPAs for affected twins and co-twins in ADHD-discordant pairs, respectively (Fig. 1a). These trends were similar for MZ twins (Fig. 1b). The most common MPAs in participants with ADHD were overweight (32%), hypermobility (30%), vision impairment (24%; 29% of these with corrective lenses), and straight eyebrows (22%). However, no association was found between ADHD and MPAs in the cohort (OR = 1.05, 95% CI = .93–1.19, p = .435), in the within-pairs analysis for all participants (OR = .53, 95% CI = .24–1.19, p = .123), or when looking at MZ twins only (OR = .36, 95% CI = .09–1.44, p = .151) (Additional file 4: Table S4). We examined the presence of MPAs in the three ADHD-discordant MZ twin pairs in our sample. No major differences in the number or types of MPAs were noted within these twin pairs (Additional file 6: Table S6).

### MPAs by zygosity

**Fig. 2:**
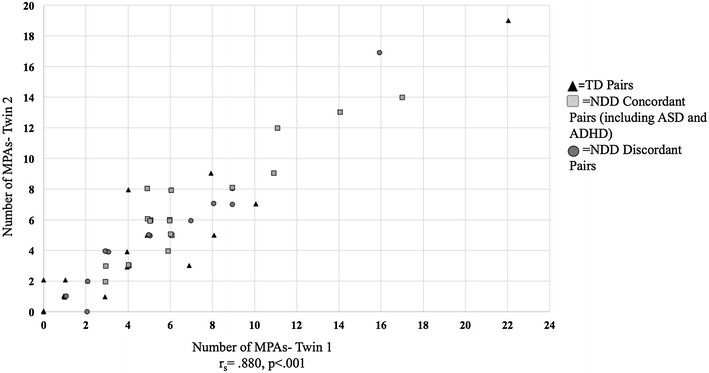
Correlation plot illustrating the association between number of MPAs within MZ twin pairs with points by TD and concordance of a diagnosis of NDD. The number of MPAs within MZ twin pairs were highly correlated [Spearman correlation (r_s_) = .88, p < .001]

The number of MPAs within MZ twin pairs was highly correlated [Spearman correlation (r_s_) = .88, p < .001]. In contrast, no correlation was seen in the DZ pairs (r_s_ = − .19, p = .676) (Fig. 2 and Additional file 7: Figure S1). MZ pairs had higher numbers of identical MPAs within pairs (Md = 4) compared to DZ pairs (Md = 1; z = − 2.764, p = .006). Additionally, we found that within MZ pairs, there was a smaller median difference in the specific MPAs present within pairs (Md = 2) compared to DZ pairs (Md = 4, z = − 1.066, p = .287).

## Discussion

This study examined MPAs in a sample of twins concordant or discordant for NDDs, as well as TD pairs. While MPAs were descriptively more frequent in individuals with NDDs, particularly those with ASD, which is in alignment with earlier studies [1, 4, 9, 10], only ASD was significantly associated with the extent of MPAs. Interestingly, the association was not only confirmed for clinical ASD, but also autistic traits. Bolton et al. [18] suggested that the presence of MPAs in individuals with ASD may point to genetic issues such as de novo copy number variants. Indeed, Miles and Hillman [1] found individuals with atypical phenotypes (as assessed through physical anomalies on morphological exams) were 10 times more likely to be diagnosed with a genetic syndrome. Tammimies et al. [10] also found that individuals with greater numbers of MPAs were more likely to receive a positive result on genetic testing through both whole exome sequencing and chromosomal microarray. Therefore, examining children for MPAs in ASD may provide insight into potential genetic issues for this neurodevelopmental disorder. Although recommendations exist within the USA to conduct genetic testing on any child who receives a diagnosis of a neurodevelopmental disorder [19, 20], similar recommendations have not yet been made in other parts of the world. Even with recommendations for genetic testing, recent studies from the USA show parents report just under 35% of children with ASD actually receive genetic testing [21]. Due to the constraints that may exist in conducting universal genetic testing for children diagnosed with ASD that are summarized in Tammimies et al. [22], an approach to the diagnostic evaluation of a child with ASD that takes into account the number of MPAs from a clinical genetic assessment as screening for a potential underlying genetic cause for the disorder may be warranted.

For participants with ADHD or any NDD diagnosis, no associations were found between MPAs and either diagnosis. For ADHD, these results are similar to previous research in children [6], but conflict with a more recent study in adults reporting an increasing number of MPAs in individuals with ADHD compared with controls [8]. Finally, we did find that IQ was significantly associated with MPAs, similar to previous research [6].

The most frequent MPAs found in individuals in our study confirmed earlier findings, for example, hypermobility/lax joints and flat feet in ASD [7]. Additionally, previous research has found overrepresentation of overweight cases in ASD and ADHD cohorts [23], similar to our study. Although our original hypothesis was that affected twins from MZ NDD-discordant pairs would have more MPAs than their co-twin, this was not found in our study. We did find the presence of scoliosis in two affected twins and not their co-twins. Scoliosis has been reported previously in children with ASD and ADHD with known genetic syndromes such as of the 16p11.2 deletion syndrome [24, 25]. In our study, a total of five participants had scoliosis (two diagnosed with ASD, one diagnosed with ASD and ADHD, and two with TD). Genetic testing is being performed on all study participants, and the results could be further explored for the presence of any high risk genetic variants in these participants.

We explored the association between the total number of MPAs and the number of specific MPAs that differed within pairs and demonstrated that MZ pairs had a stronger correlation of MPAs compared to DZ pairs. Our findings are similar to a nested case–control study of children with ASD and their siblings that found that the adjusted odds of congenital defects were not different for cases compared to sibling controls [26], whereas an older study showed that children diagnosed with ASD had more minor malformations than their siblings and matched TD children [27]. The results of our study suggest that genetic factors may strongly influence the presence of MPAs and that additional risk factors, such as non-shared environmental factors, may contribute to differences in the NDD diagnoses within the twin pairs, especially in ASD-discordant MZ pairs, as we have shown in recent studies [28, 29]. Additionally, epigenetic factors or variable expressivity of genetic syndromes, such as 16p11.2 deletion syndrome, may explain why the diagnostic phenotype varied within these discordant pairs [30].

This study addressed several weaknesses noted in previous research through the use of standardized measures for diagnoses, comprehensive MPA assessments, report of interrater agreement, and examination of the effects of IQ and gender on MPAs. Although previous research has utilized matched patient–control pairs to explore MPAs in ASD [4], this is only the second study to examine differences in MPAs in twins with ASD and the first study in twins with ADHD or NDDs overall. Our sample was comprised mainly of MZ twins, which allows for exploration of the relationship between genetics and environmental factors in the development of MPAs, as was called for in early research on MPAs and ASD [31].

Still, there are also several limitations to this study. The comparably small number of individuals with NDD diagnoses may have limited the study’s power to detect associations with MPAs for NDDs other than ASD. Moreover, there was lack of blinding of the diagnoses as the clinical geneticists needed to interact with the participants to complete the MPA assessments. However, they were not aware of the participants’ consensus diagnoses. The MPA checklist was developed specifically for this study with no previous validation, although it was based on extensive clinical expertise, along with the use of common dysmorphology references. Neither parent MPA assessments nor parent photos were obtained in the study, as was done in previous studies [1, 10]. Therefore, the familial tendency of some MPAs was not possible to ascertain. Finally, MPA assessments are subject to examiner bias. Research is currently underway in our center to explore the ability to conduct assessments using computer-based technology to potentially limit the subjectivity of these assessments.

## Conclusion

This study found that increasing numbers of MPAs are positively associated with ASD diagnoses and ASD traits, as well as IQ, although no association was found between the number of MPAs and other NDDs. This study also supports the notion that MPAs have a strong genetic basis. As previous studies point to higher numbers of MPAs in individuals with potential genetic reasons for ASD [9, 10, 32], the findings in this study point to the potential value of a MPA assessment as part of the diagnostic evaluation for an individual with ASD, which may help classify individuals for whom genetic testing should prioritized.

## Additional files

**Additional file 1: Table S1.** Description of instruments from RATSS used in this study.

**Additional file 2: Table S2.** Minor physical anomalies checklist.

**Additional file 3: Table S3.** Additional demographic findings on the sample.

**Additional file 4: Table S4.** Cross-trait, cross-twin correlation.

**Additional file 5: Table S5.** All cohort and within-pair estimates of relationship between MPAs, categorical diagnoses and dimensional variables.

**Additional file 6: Table S6.** Description of differences in MPAs found in monozygotic pairs discordant for ASD and ADHD.

**Additional file 7: Figure S1.** Correlation plot illustrating the association between number of MPAs within DZ twin pairs with points by TD and concordance of a diagnosis of NDD. No significant correlation was seen within the DZ twin pairs (r_s_ = − .19, p = .676). Note: *MPAs* minor physical anomalies, *TD* typical development, *ASD* autism spectrum disorder, *ADHD* attention–deficit/hyperactivity disorder, *NDD* neurodevelopmental disorder.

## Abbreviations

MPAs: minor physical anomalies
NDDs: neurodevelopmental disorders
MZ: monozygotic
DZ: dizygotic
ASD: autism spectrum disorder
ADHD: attention-deficit/hyperactivity disorder
ID: intellectual disability
TD: typically developing
DSM: diagnostic and statistical manual of mental disorders
ADOS-2: Autism Diagnostic Observation Schedule
ADI-R: Autism Diagnostic Interview-Revised
K-SADS: Kiddie Schedule for Affective Disorders and Schizophrenia
DIVA 2.0: Diagnostic Interview for ADHD in Adults
IQ: intelligence quotient
WAIS-IV: Wechsler Adult Intelligence Scale-IV
WISC-IV: Wechsler Intelligence Scale for Children-IV
PPVT-4: Peabody Picture Vocabulary Test
SRS-2: Social Responsiveness Scale-2
sMRI: structural magnetic resonance imaging
GEE: generalized estimating equation
OR: odds ratio
CI: confidence interval
