# Supplementary material for: Minor physical anomalies in neurodevelopmental disorders: a twin study
Source: Child Adolesc Psychiatry Ment Health. 2017 Nov 28;11:57. doi: 10.1186/s13034-017-0195-y (PMC5706157; doi:10.1186/s13034-017-0195-y)
Supplement: Supplementary file 1 — Additional file 1: Table S1. Description of instruments from RATSS used in this study. [file 13034_2017_195_MOESM1_ESM.docx]

Supplementary Table 1. Description of Instruments from RATSS Used in this Study

| Instrument | Reference | Purpose of Instrument in RATSS |
| --- | --- | --- |
| Autism Diagnostic Observation Schedule (ADOS-2) | Lord C, Rutter M, DiLavore P, Risi S, Gotham K, Bishop S. Autism Diagnostic Observation Schedule-2^nd^ Edition (ADOS-2). Los Angeles, CA: Western Psychological Services; 2012. | Diagnosis of ASD |
| Autism Diagnostic Interview-Revised (ADI-R) | Rutter M, Le Couteur A, Lord C. The Autism Diagnostic Interview-Revised (ADI-R). Los Angeles, CA: Western Psychological Services; 2003. | Diagnosis of ASD |
| Social Responsiveness Scale-2 (SRS-2) | Constantino JN. Social Responsiveness Scale (SRS). Los Angeles, CA: Western Psychological Services; 2005. | Measurement of autistic traits |
| Kiddie Schedule for Affective Disorders and Schizophrenia (K-SADS) | Kaufman J, Birmaher B, Brent D, Rao U, Flynn C, Moreci P, et al. Schedule for Affective Disorders and Schizophrenia for School-Age Children-Present and Lifetime Version (K-SADS-PL): initial reliability and validity data. J Am Acad Child Adolesc Psychiatry. 1997;36(7):980-8. | Diagnosis of ADHD in youth |
| Diagnostic Interview for ADHD in Adults (DIVA 2.0) | Kooij JJS. Diagnostic Interview for ADHD in Adults 2.0 (DIVA 2.0). Amsterdam: Pearson Assessment and Information BV; 2010. | Diagnosis of ADHD in adults |
| Wechsler Adult Intelligence Scale-IV (WAIS-IV) | Wechsler D. WAIS-IV Wechsler Intelligence Scale for Adults 4^th^ Edition: Technical and Interpretive Manual: Pearson; 2003. | IQ in adults; diagnosis of ID |
| Wechsler Intelligence Scale for Children-IV (WISC-IV) | Wechsler D. WISC-IV Wechsler Intelligence Scale for Children 4^th^ Edition Technical and Interpretive Manual: Pearson; 2003. | IQ in children; diagnosis of ID |
| Leiter International Performance Scale- Revised | Roid GH, Miller LJ. Leiter International Performance Scale-Revised: Examiner's Manual. Wood Dale, IL: Stoelting; 1997. | Non-verbal intellectual abilities; diagnosis of ID |
| Peabody Picture Vocabulary Test, Fourth Edition (PPVT-4) | Dunn LM, Dunn DM. Peabody Picture Vocabulary Test, 4th Edition. San Antonio, TX: Pearson; 2007. | Verbal intellectual abilities; diagnosis of ID |

Instruments used in RATSS for psychological and neuropsychological testing. Note: IQ=Intelligence Quotient, ASD=Autism Spectrum Disorder, ADHD=Attention–Deficit/Hyperactivity Disorder, ID=Intellectual Disability, NDD=Neurodevelopmental Disorder
