# Supplementary material for: Minor physical anomalies in neurodevelopmental disorders: a twin study
Source: Child Adolesc Psychiatry Ment Health. 2017 Nov 28;11:57. doi: 10.1186/s13034-017-0195-y (PMC5706157; doi:10.1186/s13034-017-0195-y)
Supplement: Supplementary file 2 — Additional file 2: Table S2. Minor physical anomalies checklist. [file 13034_2017_195_MOESM2_ESM.docx]

Supplementary Table 2. Minor Physical Anomalies Checklist

| **Original Checklist (Swedish)** | **Checklist translated into English (with standardized terminology)** | **Common Anomalies (removed from Final Checklist)** | **Final Checklist (MPAs)** | **Anomalies Assessed for Interrater Agreement** |
| --- | --- | --- | --- | --- |
| **Kroppsproportioner** | **Body Proportions** |  |  |  |
| Övervikt | Overweight |  | X |  |
| Undervikt | Underweight |  | X |  |
| Asymmetri | Assymetry |  | X |  |
| Hemihypertrofi | Hemihypertrophy |  | X |  |
| Hemihypotrofi | Hemihypotrophy |  | X |  |
| Patienten långvuxen | Tall stature |  | X |  |
| Patienten kortvuxen | Short stature |  | X |  |
| Rhizomel (lår och överarmar korta) | Rhizomelic (short upper arms and legs) |  | X |  |
| Mesomel (underarmar och underben korta) | Mesomelic (short forearms and legs) |  | X |  |
| Akromel (händer o fötter små) | Acromelic (small hands and feet) |  | X |  |
| Kort bål | Short trunk |  | X |  |
| Proportionerligt kortvuxen | Proportionately short stature |  |  |  |
|  |  |  |  |  |
| **Röst** | **Voice** |  |  |  |
| Röst pipig | Squeaky voice |  | X |  |
| Röst hes | Hoarse voice |  | X |  |
| Röst nasal | Nasal voice |  | X |  |
|  |  |  |  |  |
| **Leder** | **Joints** |  |  |  |
| Överrörlighet | Hypermobility |  | X |  |
| Luxationer | Dislocation |  | X |  |
| Kontrakturer | Contractures |  | X |  |
| Felställningar | Deformities |  | X |  |
|  |  |  |  |  |
| **Huvudform avvikande** | **Head Shape** |  |  |  |
| Makrocefali | Macrocephaly |  | X |  |
| Mikrocefali | Microcephaly |  | X |  |
| Vida suturer | Wide sutures |  |  |  |
| Stora fontaneller | Large fontanelles |  |  |  |
| Kraniosynostos | Craniosynostosis |  | X |  |
| Prominent bakhuvud | Prominent occiput |  | X |  |
| Platt bakhuvud | Flat occiput |  | X |  |
|  |  |  |  |  |
| **Panna** | **Forehead** |  |  |  |
| Panna hög | High forehead |  | X |  |
| Panna låg | Low forehead |  | X |  |
| Panna buktande | Bulging forehead |  | X |  |
| Panna sluttande | Sloping forehead |  | X |  |
| Panna bred | Broad forehead |  | X |  |
| Panna smal | Short forehead = low forehead |  | X |  |
| Tinningar insjunkna | Sunken temples |  | X |  |
|  |  |  |  |  |
| **Hår eller hårfäste avvikande** | **Hair or Hairline** |  |  |  |
| Hårfäste högt i nacke och panna | High anterior and posterior hairline |  | X | X |
| Hårfäste lågt i nacke och panna | Low anterior and posterior hairline |  | X | X |
| Hårfäste treflikigt | Three-peak hairline |  | X | X |
| Cows lick | Cowlick | X |  |  |
| Widows peak | Widow's Peak | X |  |  |
| Hår tjockt | Thick hair |  | X |  |
| Hår tunt | Thin hair |  | X |  |
| Hår strävt | Rough hair |  | X |  |
| Hår ljust | Bright hair |  |  |  |
| Hår mörkt | Dark hair |  |  |  |
| Hår lockigt | Curly hair |  |  |  |
| Hår rakt | Straight hair |  |  |  |
| Hår pigmentförändringar | Pigment anomalies in hair |  | X |  |
| Hirsutism | Hirsutism |  | X | X |
|  |  |  |  |  |
| **Ögonbryn/fransar avvikande** | **Eyebrow or Eyelashes** |  |  |  |
| Ögonfransar långa | Long eyelashes |  | X | X |
| Ögonfransar korta | Short eyelashes |  | X | X |
| Ögonfransar saknas | Absent eyelashes |  | X | X |
| Ögonfransar dubbla | Double eyelashes |  | X | X |
| Ögonbryn högt placerade | High placed eyebrow |  | X | X |
| Ögonbryn lågt placerade? | Low placed eyebrow |  | X | X |
| Ögonbryn raka | Straight or horizontal eyebrow |  | X | X |
| Ögonbryn bågformade | Highly arched eyebrow |  | X | X |
| Ögonbryn outvecklade lateralt | Underdeveloped eyebrow- laterally |  | X | X |
| Ögonbryn outvecklade medialt | Underdeveloped eyebrow- medially |  | X | X |
| Ögonbryn saknas | Missing eyebrow |  | X | X |
| Synofrys | Synophrys |  | X | X |
|  |  |  |  |  |
| **Naglar avvikande** | **Nails** |  |  |  |
| Naglar dysplastiska | Dysplastic nails |  | X | X |
| Naglar avvikande form | Other abberant nail form |  | X | X |
|  |  |  |  |  |
| **Hud avvikande** | **Skin** |  |  |  |
| Hud tjock | Thick skin |  | X |  |
| Hud tunn | Thin skin |  | X |  |
| Hud ljus | Light skin |  |  |  |
| Hud mörk | Dark skin |  |  |  |
| Hud torr | Dry skin | X |  |  |
| Hud fet | Oily skin |  |  |  |
| Hud sammetslen | Velvety skin |  | X |  |
| Hud åldrad | Aged skin |  | X |  |
| Hud överskott | Excess skin |  | X |  |
| Hud stram | Tight skin |  | X |  |
| Hud onormal fettdistribution | Abnormal fat distribution |  |  |  |
| Café au lait-fläckar | Café au lait spots | X (single) | X (multiple) |  |
| Hypopigmenteringar | Hypopigmentation |  | X |  |
| Nevi | Nevi | X (>0.5 cm) |  |  |
| Hemangiom | Hemangioma |  | X |  |
| Tumörer | Tumor |  | X |  |
| Blåsor | Blisters |  | X |  |
| Papler | Papules |  | X |  |
| Mamillernas antal avvikande | Abnormal mamillary number |  | X |  |
| Mamillernas placering avvikande | Abnormal mamillary placement |  | X |  |
|  |  |  |  |  |
| **Ansiktform avvikande** | **Facial Form** |  |  |  |
| Ansiktsform runt | Round face |  | X |  |
| Ansiktsform fyrkantigt | Square face |  | X |  |
| Ansiktsform triangelformat | Triangular face |  | X |  |
|  |  |  |  |  |
| **Nacke avvikande** | **Neck** |  |  |  |
| Nacke bred | Broad neck |  | X |  |
| Nacke kort | Short neck |  | X |  |
| Nacke ökat nackskinn | Redundant nuchal skin |  | X |  |
| Nacke pterygium | Neck webbing |  | X |  |
|  |  |  |  |  |
| **Ögon avvikande** | **Eyes** |  |  |  |
| Synavvikelse | Visual Impairment |  | X |  |
| Typ av synnedsättning | Type of vision impairment |  |  |  |
| Ögonen små | Microphtalmia |  | X | X |
| Ögonglob saknas | Missing eyeballs or Anophthalmia |  | X | X |
| Iris avvikelser | Iris abnormalities |  | X | X |
| Ögonen prominenta | Prominent eyes |  | X | X |
| Ögonen djupt liggande | Deeply set eyes | X |  |  |
| Hypertelorism | Hypertelorism |  | X | X |
| Hypotelorism | Hypotelorism |  | X | X |
| Ögonspringor korta | Short palpebral fissure |  | X | X |
| Ögonspringor långa | Long palpebral fissure |  | X | X |
| Ögonspringor nedåtsluttande | Downslanted palpebral fissure |  | X | X |
| Ögonspringor uppåtsluttande | Upslanted palpebral fissure |  | X | X |
| Epikantus | Epicanthus |  | X | X |
| Telekantus | Telecanthus |  | X | X |
| Ptos | Ptosis | X |  |  |
| Kolobom | Coloboma |  | X | X |
| Telangiektasier | Telangiectasias |  | X | X |
| Avvikande tårproduktion | Abnormal tear production |  |  |  |
| Ögonbottenundersökning avvikande | Abnormality in retina |  |  |  |
| Linsdislokation | Lens dislocation |  |  |  |
| Näthinneavlossning | Retinal detachment |  |  |  |
| Glaukom | Glaucoma |  |  |  |
| Katarakt | Cataract |  |  |  |
|  |  |  |  |  |
| **Munregion avvikande** | **Mouth Region** |  |  |  |
| Mun stor | Wide mouth |  | X | X |
| Mun liten | Narrow mouth |  | X | X |
| Överläppen tältformad | Tented mouth |  | X | X |
| Läpparna tjocka | Thick lip | X |  |  |
| Läpparna smala | Thin lip | X |  |  |
| Läppspalt | Cleft lip |  | X | X |
| Andra avvikelser, t.ex. gropar, upphöjningar eller avvikelser av frenulum | Other anomalies- e.g., dimples, ridges, or deviations of frenulum |  | X | X |
| Gom hög | High palate | X |  |  |
| Gom spetsig | Angled palate |  | X |  |
| Uvula bred | Broad uvula |  | X |  |
| Uvula bifid | Bifid or cleft uvula |  | X |  |
| Gingiva tjock | Thick gingiva |  | X |  |
| Tunga stor | Large tongue |  | X |  |
| Tunga grov | Furrowed tongue |  |  |  |
| Tunga missbildad | Malformed tongue |  | X |  |
| Mikrognati | Micrognathia |  | X | X |
| Prognati | Prognathia |  | X | X |
|  |  |  |  |  |
| **Filtrum avvikande** | **Philtrum** |  |  |  |
| Filtrum långt | Long philtrum |  | X | X |
| Filtrum kort | Short philtrum |  | X | X |
| Filtrum utslätat | Smooth philtrum | X |  |  |
| Filtrum djupt | Deep philtrum |  | X | X |
|  |  |  |  |  |
| **Tänder avvikande** | **Teeth** |  |  |  |
| Bett trångt | Dental crowding | X |  |  |
| Bett brett | Widely spaced teeth | X |  |  |
| Tänder glesa | Oligodontia |  | X |  |
| Tänder stora | Macrodontia |  | X |  |
| Tänder små | Microdontia |  | X |  |
| Avvikande tandform | Abnormal tooth shape |  | X |  |
| Emaljdefekter | Enamel defect |  | X |  |
| Hypodonti | Hypodontia |  | X |  |
| Adonti | Anodontia |  | X |  |
| Extra tänder | Additional teeth |  | X |  |
| Onormal tid för tanderuption | Delayed or advanced eruption |  | X |  |
| Andra tandavvikelser | Other dental abnormalities |  | X |  |
|  |  |  |  |  |
| **Öron avvikande** | **Ears** |  |  |  |
| Små | Microtia |  | X | X |
| Stora | Long ear |  | X | X |
| Dysplastiska | Dysplastic ears |  | X | X |
| Lågt sittande | Low-set ear |  | X | X |
| Bakåtroterade | Posteriorly rotated ear |  | X | X |
| Helices nervikta | Overfolded helix | X |  |  |
| Helices tjocka | Thick helices |  | X | X |
| Helices tunna | Thin helices |  | X | X |
| Örsnibben avvikande form | Abnormal earlobe form | X |  |  |
| Preaurikulära bihang, gropar/fåror | Preauricular tags or pits |  | X | X |
| Hörselnedsättning | Hearing loss |  |  |  |
| Hörselnedsättning typ | Hearing loss type |  |  |  |
|  |  |  |  |  |
| **Näsa avvikande** | **Nose** |  |  |  |
| Näsa liten | Small nose | X |  |  |
| Näsa stor | Prominent Nose |  | X | X |
| Näsa lång | Long nose |  | X | X |
| Näsa kort | Short nose | X |  |  |
| Näsa platt | Flat nose |  | X | X |
| Näsa nedåtböjd | Hooked nose |  | X | X |
| Näsa uppåtböjd | Upturned tip of the nose |  | X | X |
| Näsrygg hög | High nasal bridge | X |  |  |
| Näsrygg låg | Low nasal bridge |  | X | X |
| Näsrygg bred | Broad nasal bridge | X |  |  |
| Näsrygg smal | Thin nasal bridge |  | X | X |
| Nästipp bred | Broad nasal tip |  | X | X |
| Nästipp smal | Narrow nasal tip |  | X | X |
| Nässkiljevägg kort | Short columella |  | X | X |
| Nässkiljevägg slutar den nedanför näsvingen | Low hanging columella | X |  |  |
| Näsvingar små | Narrow naris |  | X | X |
| Näsvingar anteverterade | Anteverted nares | X |  |  |
| Koanalatresi | Choanal atresia |  |  |  |
| Anosmi | Anosmia |  |  |  |
|  |  |  |  |  |
| **Axlar och armar avvikande** | **Shoulders and Arms** |  |  |  |
| Axlar avvikande | Abnormal shoulders |  | X |  |
| Överarm avvikande | Abnormal upper arm |  | X |  |
| Underarm avvikande | Abnormal forearm |  | X |  |
|  |  |  |  |  |
| **Händer avvikande** | **Hands** |  |  |  |
| Tummar lågt ansatta | Proximally placed thumbs |  | X |  |
| Tummar subluxerade | Sublaxed thumbs |  |  |  |
| Tummar breda | Broad thumbs |  | X | X |
| Tummar saknas | Missing thumbs |  | X | X |
| Tummar små | Small thumbs |  | X | X |
| Tummar trifalangeala | Triphalangeal thumbs |  | X | X |
| Klinodaktyli | Clinodactyly |  | X | X |
| Polydaktyli | Polydactyly |  | X | X |
| Syndaktyli | Syndactyly |  | X | X |
| Ektrodaktyli | Ectrodactyly |  | X | X |
| Araknodaktyli | Slender or narrow fingers |  | X | X |
| Brakydaktyli | Short fingers |  | X | X |
| Fyrfingerfåra | Single transverse palmar crease |  | X | X |
| Kuddiga fingertoppar | Fetal fingertip pads | X |  |  |
| Ödem | Edema |  | X | X |
| Avvikande mönster eller fåror i handflatorna | Abnormal patterns or groves in palms |  | X | X |
|  |  |  |  |  |
| **Höfter och ben avvikande** | **Hips and Bones** |  |  |  |
| Höfter avvikande | Abnormal hips |  | X | X |
| Lår avvikande | Abnormal thighs |  | X | X |
| Underben avvikande | Abnormal leg |  | X | X |
|  |  |  |  |  |
| **Fötter avvikande** | **Feet** |  |  |  |
| Fötterna små | Short foot |  | X | X |
| Fötterna stora | Long foot |  | X | X |
| Fötterna smala | Narrow foot |  | X | X |
| Fötterna breda | Broad foot |  | X | X |
| Polydaktyli | Polydactyly |  | X | X |
| Syndaktyli | Syndactyly |  | X | X |
| Ektrodactyli | Ectrodactyly |  | X | X |
| Araknodaktyli | Slender or long toes |  | X | X |
| Brakydaktyli | Short toes |  | X | X |
| Prominenta hälar | Prominent heel |  | X | X |
| Breda tår | Broad toes |  | X | X |
| Platta fötter | Pes planus |  | X | X |
| Sandal gap | Sandal gap | X |  |  |
| Ödem | Edema |  | X | X |
|  |  |  |  |  |
| **Rygg avvikande** | **Back** |  |  |  |
| Skolios | Scoliosis |  | X |  |
| Kyfos | Kyphosis |  | X |  |
|  |  |  |  |  |
| **Buk avvikande** | **Abdomen** |  |  |  |
| Navelbråck | Umbilical hernia |  | X |  |
| Ljumskbråck | Inguinal hernia |  | X |  |
| Rectusdiastas | Rectus diastasis |  | X |  |
|  |  |  |  |  |
| **Yttre genitalia avvikande** | **Outer genitalia** |  |  |  |
| Kryptorkidism | Cryptorchidism |  | X |  |
| Hypospadi | Hypospadias |  | X |  |
| Mikropenis | Micropenis |  | X |  |
| Bifid skrotum | Bifid scrotum |  | X |  |
| Shawl skrotum | Shawled scrotum |  | X |  |
| Testiklarna små | Small testes |  | X |  |
| Testiklarna stora | Large tests |  | X |  |
| Andra missbildningar | Other malformations |  | X |  |

Checklist used for MPA assessment.
