# Supplementary material for: Minor physical anomalies in neurodevelopmental disorders: a twin study
Source: Child Adolesc Psychiatry Ment Health. 2017 Nov 28;11:57. doi: 10.1186/s13034-017-0195-y (PMC5706157; doi:10.1186/s13034-017-0195-y)
Supplement: Supplementary file 3 — Additional file 3: Table S3. Additional demographic findings on the sample. [file 13034_2017_195_MOESM3_ESM.docx]

Supplementary Table 3: Additional Demographic Findings on the Sample

| Characteristic |  |
| --- | --- |
| Mother Country of Origin (by twin pairs) | **N (%)** |
| Sweden | 48 (82.8) |
| Finland | 4 (6.9) |
| Chile | 1 (1.7) |
| Dominican Republic | 1 (1.7) |
| Hungary | 1 (1.7) |
| Germany | 1 (1.7) |
| USA | 1 (1.7) |
| Unknown | 1 (1.7) |
| Father Country of Origin (by twin pairs) |  |
| Sweden | 46 (79.3) |
| Finland | 5 (8.6) |
| Chile | 1 (1.7) |
| Denmark | 1 (1.7) |
| Dominican Republic | 1 (1.7) |
| England | 1 (1.7) |
| Gambia | 1 (1.7) |
| Turkey | 1 (1.7) |
| Unknown | 1 (1.7) |

Birth country of the mother and father of the study twins (race/ethnicity data not collected on participants per national standards).
