# Supplementary material for: Minor physical anomalies in neurodevelopmental disorders: a twin study
Source: Child Adolesc Psychiatry Ment Health. 2017 Nov 28;11:57. doi: 10.1186/s13034-017-0195-y (PMC5706157; doi:10.1186/s13034-017-0195-y)
Supplement: Supplementary file 4 — Additional file 4: Table S4. Cross-trait, cross-twin correlation. [file 13034_2017_195_MOESM4_ESM.docx]

Supplementary Table 4: Cross-Trait, Cross-Twin Correlation

| Comparison | Correlation |
| --- | --- |
| Total MPA score of one twin with total raw SRS score of co-twin | Beta: 3.34  (95% CI= 1.39-5.30), p<.001 |
| Total MPA score of one twin with IQ of co-twin | Beta: -.88  (95% CI=-1.52- -.24), p=.007 |

Cross-trait associations between the MPA score in one twin with the total raw SRS-2 score or IQ for the other twin to explore the genetic correlation of the measures.
