# Supplementary material for: Minor physical anomalies in neurodevelopmental disorders: a twin study
Source: Child Adolesc Psychiatry Ment Health. 2017 Nov 28;11:57. doi: 10.1186/s13034-017-0195-y (PMC5706157; doi:10.1186/s13034-017-0195-y)
Supplement: Supplementary file 5 — Additional file 5: Table S5. All cohort and within-pair estimates of relationship between MPAs, categorical diagnoses and dimensional variables. [file 13034_2017_195_MOESM5_ESM.docx]

Supplementary Table 5: All Cohort and Within-Pair Estimates of Relationship Between MPAs, Categorical Diagnoses and Dimensional Variables

|  |  | Cohort Estimate | | | | Within-Pair Conditional Estimate | | | | MZ Only Within-Pair Conditional Estimate | | | |
| --- | --- | --- | --- | --- | --- | --- | --- | --- | --- | --- | --- | --- | --- |
| Categorical Diagnoses |  | Beta | OR | 95% CI (OR) | p-value | Beta | OR | 95% CI (OR) | p-value | Beta | OR | 95% CI (OR) | p-value |
| ASD and MPAs | No Adjustments | 0.25 | 1.29 | 1.00 − 1.66 | .047 | 0.25 | 1.28 | .60 − 2.75 | .529 | 0.35 | 1.42 | 0.63 − 3.19 | .398 |
|  | Adjusted for IQ | 0.23 | 1.26 | .98 − 1.61 | .073 | 0.16 | 1.18 | .55 − 2.54 | .673 | 0.55 | 1.73 | .47 − 6.45 | .413 |
|  | Adjusted for ADHD | 0.25 | 1.29 | 1.02 − 1.63 | .032 | 1.00 | 2.71 | .84 − 8.80 | .097 | 1.00 | 2.71 | .83 − 8.81 | .097 |
|  | Adjusted for NDDs | 0.28 | 1.33 | 1.05 − 1.67 | .016 | 1.00 | 2.71 | .84 − 8.80 | .097 | 1.00 | 2.71 | .83 − 8.81 | .097 |
| ADHD and MPAs | No Adjustments | 0.05 | 1.05 | .93 − 1.19 | .435 | −0.63 | 0.53 | .24 − 1.19 | .123 | −1.01 | 0.36 | .09 − 1.44 | .151 |
|  | Adjusted for IQ | 0.03 | 1.03 | .91 − 1.17 | .625 | −1.02 | 0.36 | .09 − 1.42 | .145 | −1.05 | 0.35 | .07 − 1.80 | .209 |
|  | Adjusted for ASD | −0.03 | 0.98 | .87 − 1.09 | .665 | −0.26 | 0.77 | .33 − 1.80 | .550 | −0.42 | 0.66 | .14 − 3.13 | .598 |
|  | Adjusted for NDDs | −0.01 | 1.00 | .89 − 1.12 | .929 | −0.60 | 0.55 | .25 − 1.19 | .130 | −0.42 | 0.66 | .14 − 3.13 | .598 |
| NDDs and MPAs | No Adjustments | 0.09 | 1.09 | .94 − 1.27 | .256 | 0.10 | 1.10 | .46 − 2.65 | .832 | 0.11 | 1.11 | .44 − 2.82 | .824 |
|  | Adjusted for IQ | 0.05 | 1.05 | .89 − 1.24 | .592 | −0.09 | 0.91 | .37 − 2.27 | .845 | −0.45 | 0.64 | .26 − 1.57 | .326 |
| Dimensional Traits |  | Beta | SE | 95% CI (Beta) | p-value | Beta | SE | 95% CI (Beta) | p-value | Beta | SE | 95% CI (Beta) | p-value |
| IQ and MPAs | No Adjustments | −0.95 | 0.32 | −1.59 − −.32 | .003 | 0.30 | 0.69 | −1.04 − 1.64 | .665 | −0.15 | 0.67 | −1.46 − 1.17 | .828 |
| SRS-2 and MPAs | No Adjustments | 3.02 | 0.98 | 1.09 − 4.94 | .002 | 1.49 | 1.80 | −2.04 − 5.02 | .409 | 2.11 | 1.85 | −1.51 − 5.73 | .254 |
|  | Adjusted for IQ | 2.28 | 0.97 | .37 − 4.18 | .019 | 1.78 | 1.83 | −1.82 − 5.37 | .332 | 1.88 | 1.68 | −1.41 − 5.17 | .263 |

All cohort and within-pairs GEE analyses of association between the number of MPAs and either categorical or dimensional diagnoses, including adjustments for IQ and diagnoses. Note: MPAs=Minor Physical Anomalies, MZ=Monozygotic, IQ=Intelligence Quotient, ASD=Autism Spectrum Disorder, ADHD=Attention–Deficit/Hyperactivity Disorder, ID=Intellectual Disability, NDD=Neurodevelopmental Disorder, SRS-2= Social Responsiveness Scale-2
