# Supplementary material for: Minor physical anomalies in neurodevelopmental disorders: a twin study
Source: Child Adolesc Psychiatry Ment Health. 2017 Nov 28;11:57. doi: 10.1186/s13034-017-0195-y (PMC5706157; doi:10.1186/s13034-017-0195-y)
Supplement: Supplementary file 6 — Additional file 6: Table S6. Description of differences in MPAs found in monozygotic pairs discordant for ASD and ADHD. [file 13034_2017_195_MOESM6_ESM.docx]

Supplementary Table 6: Description of Differences in MPAs Found in Monozygotic Pairs Discordant for ASD and ADHD

| Discordant Twin Pairs by Diagnosis | Number of MPAs for Affected Twin | Number of MPAs for Co-Twin | Number of Concordant MPAs | Number and Type of MPAs of Discordance |
| --- | --- | --- | --- | --- |
| ASD-1 | 8 | 7 | 4 | Proband (4): Body asymmetry, hip and thigh abnormalities, scoliosis  Co-Twin (3): Short trunk, hypermobility in joints, micrognathia/prognathia |
| ASD-2 | 4 | 3 | 3 | Proband (1): Underweight |
| ASD-3 | 17 | 16 | 14 | Proband (3): Scoliosis, inguinal hernia, other genital abnormality  Co-Twin (2): Overweight, straight eyebrows |
| ASD-4 | 9 | 7 | 7 | Proband (2): Macrocephaly, abnormally placed mamillaries |
| ASD-5 | 6 | 8 | 3 | Proband (2): Hypopigmented skin, abnormal shoulders  Co-Twin (5): Overweight, hypertelorism, long palpebral fissure, microtia, pre-auricular tags/pits |
| ASD-6 | 9 | 8 | 8 | Proband (1): Hemangioma |
| ASD-7 | 6 | 7 | 5 | Proband (1): Vision impairment  Co-Twin (2): Underweight, triangular face |
| ADHD-1 | 1 | 1 | 1 | N/A |
| ADHD-2 | 6 | 6 | 6 | N/A |
| ADHD-3 | 0 | 2 | 0 | Co-Twin (2): Café au Lait spot, round face |

Comparison of pairs discordant for a diagnosis of ASD or ADHD. The table shows the number of MPAs overall and the number of concordant and discordant MPAs in the affected twin and their co-twin. Note: MPAs=Minor Physical Anomalies, ASD=Autism Spectrum Disorder, ADHD=Attention–Deficit/Hyperactivity Disorder
