# Supplementary figures and images for: Minor physical anomalies in neurodevelopmental disorders: a twin study
Source: Child Adolesc Psychiatry Ment Health. 2017 Nov 28;11:57. doi: 10.1186/s13034-017-0195-y (PMC5706157; doi:10.1186/s13034-017-0195-y)

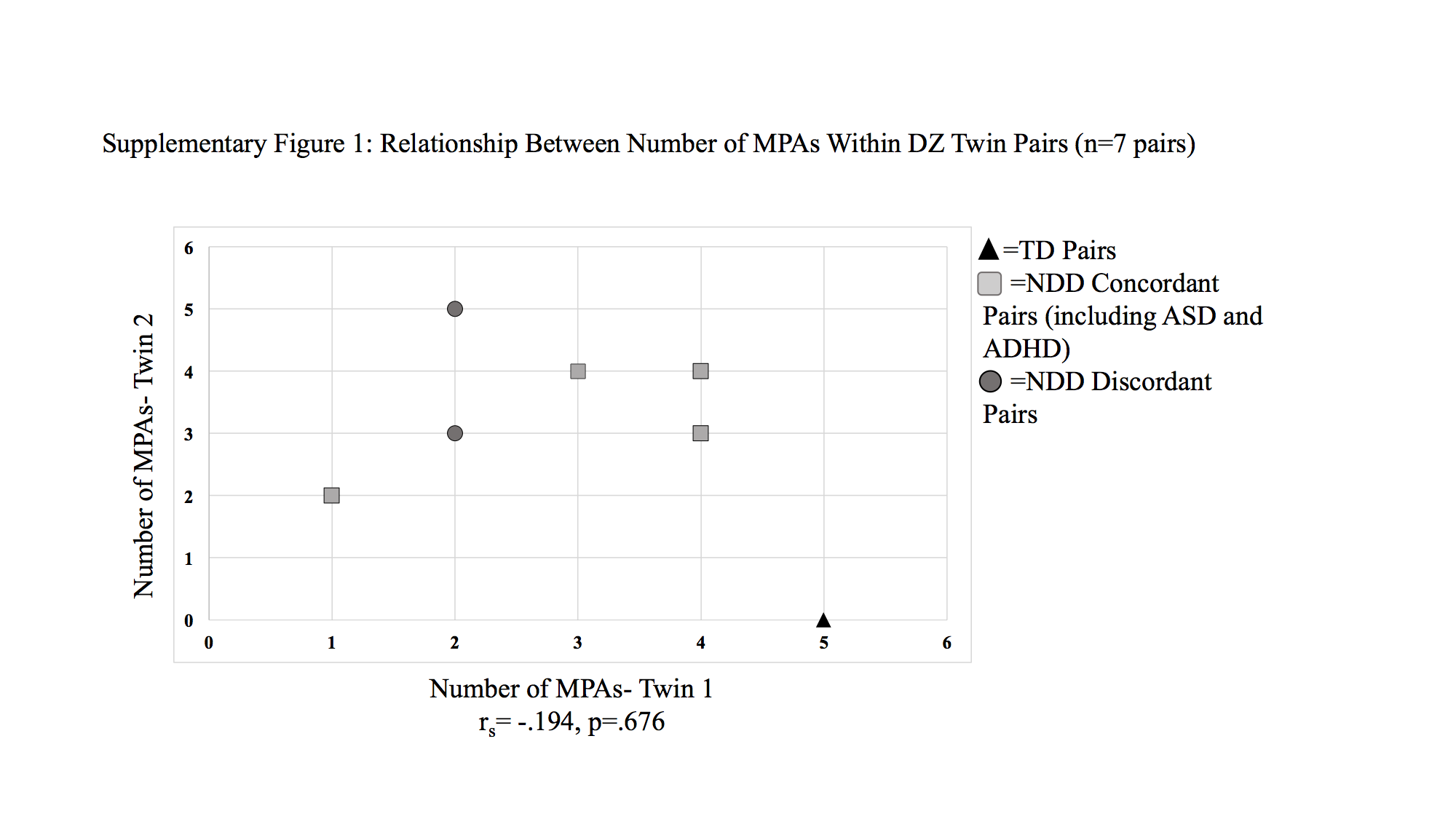

Supplement: Supplementary file 7 — Additional file 7: Figure S1. Correlation plot illustrating the association between number of MPAs within DZ twin pairs with points by TD and concordance of a diagnosis of NDD. No significant correlation was seen within the DZ twin pairs (rs = − .19, p = .676). Note: MPAs minor physical anomalies, TD typical development, ASD autism spectrum disorder, ADHD attention–deficit/hyperactivity disorder, NDD neurodevelopmental disorder. [file 13034_2017_195_MOESM7_ESM.tiff]
